# Acute myeloid leukemia with leukemic pleural effusion and high levels of pleural adenosine deaminase: A case report and review of literature

**DOI:** 10.1515/med-2021-0243

**Published:** 2021-03-12

**Authors:** Sing-Ting Wang, Chieh-Lung Chen, Shih-Hsin Liang, Shih-Peng Yeh, Wen-Chien Cheng

**Affiliations:** Division of Hematology and Oncology, Department of Internal Medicine, China Medical University Hospital, Taichung, Taiwan; Division of Pulmonary and Critical Care Medicine, Department of Internal Medicine, China Medical University Hospital, Taichung, Taiwan; Division of Pulmonary and Critical Care Medicine, Department of Internal Medicine, China Medical University Hospital, No. 2, Yude Road, Taichung 404, Taiwan

**Keywords:** acute myeloid leukemia, leukemic pleural effusion, adenosine deaminase, extramedullary

## Abstract

Pleural effusions are rarely observed in association with acute myeloid leukemia (AML), and their true incidence remains unknown. Given the low diagnostic yield from cytopathologic analysis of malignant pleural effusions and the fact that patients with leukemia are often thrombocytopenic and unable to tolerate invasive procedures, the incidence of leukemic effusions may be underestimated. Here, we report a rare case of pleural effusion in a patient with newly diagnosed AML. Initial analysis revealed an exudative, lymphocyte-predominant effusion. High levels of adenosine deaminase (ADA) were detected in pleural fluid, consistent with a diagnosis of tuberculosis. However, the analysis of pleural cytology revealed leukemic cells, permitting the diagnosis of leukemic effusion to be made. The patient underwent induction chemotherapy and pleural effusion resolved without recurrence. This case emphasizes the diagnostic dilemma presented by high levels of ADA in a leukemic pleural effusion, as this association has not been previously considered in the literature.

## 1 Introduction

Nearly, all hematologic malignancies can present with pleural effusions. Among the most common diseases associated with this complication are Hodgkin lymphoma and non-Hodgkin lymphoma because the mediastinum is a typical site of primary disease [1]. Leukemic pleural effusions are observed rarely only in patients with leukemia and even more infrequently among those diagnosed with acute myeloid leukemia (AML). The true incidence of leukemic effusion is not known, but it is believed to be underestimated; this complication may become more common with improved supportive care and prolonged survival for patients with AML [2].

In patients with leukemia, other causes of the pleural effusion such as infections, other disseminated solid tumors, and/or treatment-associated toxicities should be excluded [3]. The results from immunocytologic examination, as well as flow cytometry and polymerase chain reaction methodologies applied to cytology specimens, can contribute to the differential diagnosis [4].

Leukemic pleural effusion has been considered to be a reflection of the severity of the underlying hematologic malignancy; most cases have been associated with a poor prognosis [3,5,6]. The treatment of the primary disease usually results in resolution of the pleural effusion [1]; survival relies on an appropriate response to the treatment [2,3].

Adenosine deaminase (ADA) is an enzyme involved in the proliferation and differentiation of lymphocytes, especially T lymphocytes. The two principal isoenzymes are ADA1 and ADA2 [7]. While ADA1 can be detected in all cells, ADA2 is found only in macrophages and monocytes [8]. The presence of live intracellular microorganisms stimulates the release of ADA [9]. Currently, well-established evidence suggests that ADA levels >40 U/L in lymphocytic pleural effusions can be used as virtually diagnostic for tuberculous pleural effusion (TPE); this is especially true in the regions of high disease prevalence [10]. ADA levels in nontuberculous lymphocytic effusions seldom exceed the diagnostic cutoff for TPE [11]. Conversely, similar or higher ADA levels have occasionally been reported in parapneumonic effusion [12]; furthermore, empyema or lymphoma should be considered in cases with extremely high ADA activity [13]. In one retrospective study that evaluated 156 patients with malignant pleural effusion, ADA levels >40 U/L were detected in only 16 patients (1%) and none were related to acute leukemia [14].

## 2 Case report

**Figure 1:**
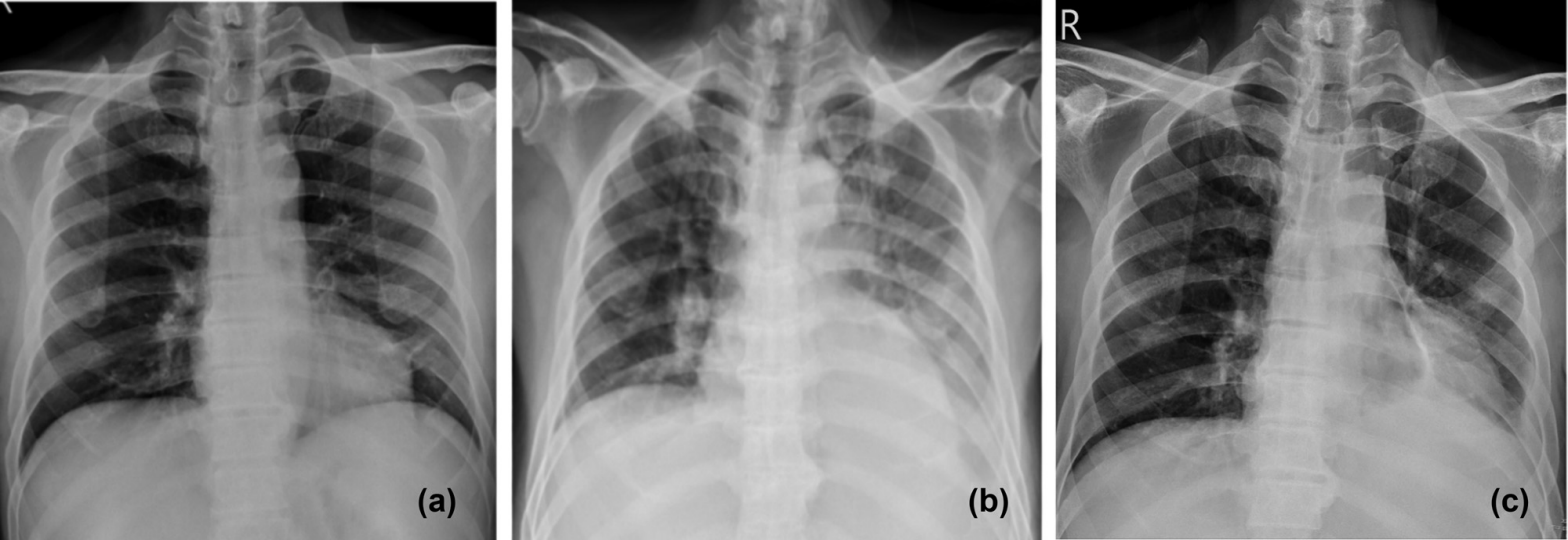
(a) Initial chest radiograph revealed no abnormal findings. (b) Chest radiograph taken revealed rapid accumulation of a left side pleural effusion within 1 week of admission. (c) Pleural effusion largely resolved after induction chemotherapy.

**Figure 2:**
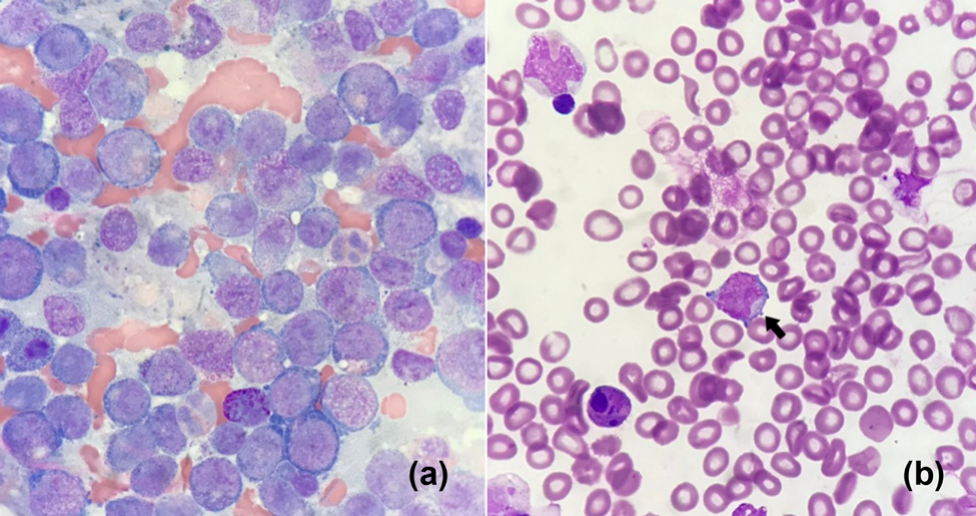
(a) Bone marrow aspiration revealed a hypercellular marrow with diffuse infiltration of myeloblasts with vesicular nuclei and scanty cytoplasm. (b) Pleural fluid cytology (cell block) was notable for the presence of atypical cells with enlarged and hyperchromatic nuclei and scant cytoplasm (arrow).


A 55-year-old man with no medical history presented with a chief complaint of dyspnea on exertion for a period of 1 month. His body temperature was 37.4°C, pulse rate was 105 beats per minute, blood pressure was 111/63 mm Hg, respiratory rate was 18 breaths per minute, and oxygen saturation was 98% with ambient air. Physical examination was normal except for pallor. Chest radiograph (Figure 1a) and electrocardiogram were normal at presentation. A complete blood count revealed leukocytosis (white blood cell [WBC] count, 97,600/µL) with 26% blasts and an elevated fraction of circulating monocytic cells (45%), anemia (hemoglobin 6.6 g/dL), and thrombocytopenia (platelet count, 23,000/µL). Other laboratory test results are presented in Table 1. Bone marrow examination was notable for hypercellularity with increased myeloblasts (Figure 2a). Immunophenotyping of bone marrow cells by flow cytometry revealed that cells were positive for CD13, CD123, CD7, CD34, CD117, and HLA-DR, but not for CD56 or terminal deoxynucleotidyl transferase (Tdt). A cytogenetic study revealed an abnormal karyotype of 47, XY, +21. e8e2(e11e3); a histone-lysine *N*-methyltransferase 2A (KMT2A)-partial tandem duplication (PTD) fusion was detected by a real-time reverse transcriptase-polymerase chain reaction (RT-PCR). A diagnosis of AML with e11e3(e8e2) MLL-PTD was made. However, the patient developed direct type hyperbilirubinemia (Table 1) and progressive shortness of breath during hospitalization; chest radiograph revealed rapid growth of left side pleural effusion (Figure 1b). Abdominal sonography showed no evidence of mechanical obstruction. Subsequently, 650 mL of a bloody effusion was withdrawn through ultrasound-guided thoracentesis. Laboratory analysis of the pleural fluid indicated an exudative, lymphocyte-predominant effusion (red blood cells, 21,692/L; WBCs, 2,025/L with a leukocyte differential including 46% lymphocytes, 38% monocytes, and 16% neutrophils; protein <3 mg/dL, glucose 84 mg/dL, and lactate dehydrogenase [LDH] 922 U/L). A high level of ADA (42 U/L) was also detected in pleural fluid. Given these findings, a diagnosis of tuberculous pleural effusion was considered. Nevertheless, rapid growth of tuberculous pleural effusion is relatively uncommon. Antituberculosis agents were not prescribed because of the diagnostic uncertainty and hyperbilirubinemia. MTB quantitative PCR (Cepheid Xpert MTB/RIF TEST with real-time PCR) of the pleural fluid yielded negative results. Chest computed tomography showed no evidence of pulmonary tuberculosis, mass lesion, or pulmonary embolism. However, cytologic examination of the pleural fluid revealed some abnormally large cells with fine chromatin and scant cytoplasm; the morphological features of these cells resembled those of the myeloblasts in the bone marrow (Figure 2b). Therefore, the diagnosis of AML with leukemic pleural effusion and suspected liver involvement was considered. The patient was treated with induction chemotherapy that included Idarubicin (12 mg/m^2^ from days 1 to 3) and Cytarabine (100 mg/m^2^ from days 1 to 7). Follow-up chest radiograph on day 7 of induction chemotherapy revealed significant resolution of the pleural effusion, and laboratory test results showed alleviation of hyperbilirubinemia (Table 1). The patient developed neutropenic fever after chemotherapy and underwent several courses of broad-spectrum antibiotic treatment. Bacterial cultures were negative in all sterile sites. Bone marrow examination on day 14 revealed significant cytoreduction with a low percentage of residual blasts. There was no recurrence of the pleural effusion (Figure 1c); culture of the pleural fluid was negative for *Mycobacterium tuberculosis*. Subsequently, the patient experienced a relapse and developed refractory disease in the clinical course and eventually died of septic shock ∼4 months after the diagnosis. Nevertheless, there was no recurrence of left side pleural effusion throughout the clinical course.

**Table 1:** Laboratory parameters

Variable	Reference range	On admission	Day 7 of hospitalization	Day 7 of induction chemotherapy
White blood cell count (per μL)	3,600–11,200	97,600	41,500	5,900
**Differential count (%)**				
Neutrophils	43.4–76.6	9	22.9	53.6
Lymphocytes	16–43.5	7	1.8	20.5
Monocytes	4.5–12.5	45	63.5	25.9
Eosinophils	0.6–7.9	0	0	0
Atypical lymphocytes	—	5	—	—
Hemoglobin (g/dL)	13.7–17	6.6	9.1	7.9
Platelet count (per μL)	1,30,000–4,00,000	23,000	15,000	<10,000
Prothrombin time (s)	9.5–11.7	13.8	—	15.7
Prothrombin time international normalized ratio	0.9–1.2	1.37	—	1.57
Activated partial thromboplastin time (s)	24.3–32.7	30.7	—	33.3
Sodium (mmol/L)	135–147	137	139	131
Potassium (mmol/L)	3.5–4.5	3.0	3.1	4.6
Urea nitrogen (mg/dL)	7–25	18	16	31
Creatinine (mg/dL)	0.7–1.3	1.6	1.11	0.61
Uric acid (mg/dL)	4.4–7.6	11.4	2.8	3.5
Lactate dehydrogenase (U/L)	140–271	419	646	157
Alanine aminotransferase (U/L)	5–40	11	15	45
Aspartate aminotransferase (U/L)	13–39	13	18	8
Total bilirubin (mg/dL)	0.2–1.3	1.14	5.21	2.24
Direct bilirubin (mg/dL)	0–0.4	—	3.34	—
Alkaline phosphatase (U/L)	38–126	—	70	64
C-Reactive protein (mg/dL)	<1.0	—	24.35	0.34
Procalcitonin (ng/mL)	<0.5	—	2.5	—

**Informed consent:** Written informed consent was obtained from the patient for publication of this case report and any accompanying images.

## 3 Discussion

Leukemic effusion, similar to other extramedullary manifestations, can develop simultaneously with or precede bone marrow involvement. Pleural effusions have been reported to be associated with different phases of AML, including at the time of initial diagnosis, during advanced refractory disease, upon relapse, or after stem cell transplantation; it may even be an isolated finding after bone marrow remission [15,16] (Table 2). Some studies report that the development of pleural effusion may serve as an indicator of the development of AML in patients with myelodysplastic syndrome [17,18].

**Table 2:** Summary of case reports of AML pleural effusion^a^

Year	Author (Ref.)	Age (years)/sex	How the diagnosis was achieved	Pleural ADA	Leukemia status at leukemic effusion diagnosis	Treatment	Outcome
1955	Raynolds et al. [34]	26/M	Cytologic examination, necropsy	Not reported	AML, newly diagnosed	Supportive care	Death 5 months after diagnosis
1974	Wu and Burns [15]	67/F	Cytologic examination	Not reported	AML, in BM remission	COAP therapy, intrapleural araC, radiation therapy	Effusion resolved
1989	Green et al. [35]	59/M	Cytologic examination	Not reported	AML, M1, newly diagnosed	Chemotherapy with mitozantrone and araC	CR with the resolution of effusion after the first course of chemotherapy
1994	Ohe et al. [36]	51/M	Not reported	Not reported	CD7 + AML, newly diagnosed	Induction araC + Daunorubicin, then autologous HSCT	CR for minimum 8 months
1996	Schmetzer et al. [37]	49/F	Cytologic examination	Not reported	AML, M5b, relapse	Induction chemotherapy, then daunomycin and araC containing consolidation	Second CR; died 6 years after diagnosis during fourth relapse of AML
2002	Park et al. [38]	41/M	Cytologic examination with cytogenetic confirmation	Not reported	AML, M2, s/p HSCT, with isolated pleural relapse 31 months after HSCT, in BM remission	Chemotherapy	Died of septicemia while undergoing chemotherapy
2003	Azoulay et al. [39]	19/F	By clinical history	Not reported	AML, M5, newly diagnosed	Chemotherapy	Died, respiratory status deteriorated and cardiac arrest developed after chemotherapy
2003	Azoulay et al. [39]	45/F	By clinical history	Not reported	AML, M5, newly diagnosed	Chemotherapy	Died, respiratory status deteriorated after chemotherapy
2003	Azoulay et al. [39]	50/M	By clinical history	Not reported	AML, M5, newly diagnosed	Chemotherapy	Alive, respiratory failure developed after chemotherapy
2003	Disel et al. [40]	39/M	Pleural biopsy	Not reported	APL, with pleural relapse	Chemotherapy with FLAG-IDA regimen	CR with resolution of pulmonary signs and symptoms
2004	Khan et al. [17]	71/F	Cytologic examination	Not reported	MDS with transformation to AML M5	Induction araC + Daunorubicin	Died of overwhelming sepsis 22 days after the initiation of induction chemotherapy
2005	Farray et al. [41]	45/F	Cytologic examination, flow cytometry	Not reported	Acute megakaryoblastic leukemia, M7, newly diagnosed	Not reported	Not reported
2005	Leong et al. [42]	25/M	Cytologic examination	Not reported	AML, M4Eo, newly diagnosed	Induction araC + Daunorubicin, re-induction with high dose araC due to refractory disease	Refractory disease, death 8 weeks after diagnosis
2007	Fatih et al. [43]	50/M	Cytologic examination, flow cytometry	Not reported	AML, M1, newly diagnosed	3 + 7 induction with Idarubicin + araC, then FLAG-IDA regimen due to refractory and old age	Effusion resolved without recurrence, but leukemia was refractory; death 3 months after discharge
2008	Huang et al. [18]	56/F	Cytologic examination	Not reported	CMMoL with transformation to AML	3 + 7 induction with Idarubicin + araC	Respiratory failure; died on day 64 during hospitalization
2008	Raina et al. [44]	22/M	Cytologic examination	Not reported	AML, M4, newly diagnosed	Chemotherapy	Died on day 3 of initiating treatment
2009	Rigamonti et al. [45]	52/M	Cytologic examination, cytogenetic study	Not reported	AML, newly diagnosed	Induction chemotherapy with EMA protocol	Died of septic emboli with ICH 4 weeks after initiation of induction chemotherapy
2011	Stoll et al. [46]	54/M	Cytologic examination, flow cytometry	Not reported	AML–MDS with erythroid differentiation, refractory to chemotherapy	None, ineligible due to renal function	Home hospice; death 6 months after diagnosis
2011	Ou et al. [16]	53/M	Cytologic examination, flow cytometry	Not reported	AML, newly diagnosed	3 + 7 induction with Idarubicin + araC, re-induction with high-dose araC due to refractory pleural effusion	CR with first induction, but effusions did not resolve. After re-induction with high-dose Cytarabine, effusions resolved. Later underwent HSCT, remained disease free for minimum 1 year
2012	Nieves-Nieves et al. [47]	66/M	Cytologic examination	Not reported	AML, newly diagnosed	3 + 7 induction with Idarubicin + araC, re-induction with EMA protocol due to refractory disease	Effusion resolved without recurrence, but leukemia was refractory; death through complications of leukemia
2013	Chang [2]	74/F	Cytologic examination	Not reported	AML, M4, newly diagnosed	Induction araC and four cycles of postremission chemotherapy with araC and etoposide	Effusions resolved; CR for minimum 11 months
2013	Chang [2]	75/M	Cytologic examination	Not reported	CMMoL with transformation to AML M4	None, ineligible due to poor performance status	Death 1 month after diagnosis
2013	Chang [2]	74/M	Cytologic examination	Not reported	AML, M4, refractory	Induction araC and subsequently four different regimens due to refractory disease	Death 1 month after diagnosis
2013	Agrawa [48]	45/M	Cytologic examination	Not reported	AML, M2, newly diagnosed	Chemotherapy	Death 1 week after diagnosis
2013	Oka et al. [49]	63/F	Cytologic examination, flow cytometry	Not reported	AML without maturation, with CD56 expression, newly diagnosed	Not reported	Death 11 month after diagnosis
2014	Duhan et al. [50]	26/F	Cytologic examination	Not reported	AML, M4, newly diagnosed	Induction chemotherapy with Daunorubicin, araC, and cladribine	Died of refractory disease
2014	Morell-García et al. [51]	76/M	Cytologic examination, flow cytometry	Not reported	AML, during the treatment	5-Azacitidine	Death 15 days after diagnosis
2014	Pemmaraju et al. [33]	55/M	Cytogenetic study^b^	Not reported	Progression of PV to AML	Decitabine for 5 days then BIDFA for 4 days	Relapsed prior to stem cell transplant; died 11 months after transformation to AML
2014	Hanenberg and Marionneaux [52]	64/F	Cytologic examination	Not reported	Progression of PV to AML	Induction araC + Daunorubicin, replaced with decitabine and ruxolitinib	Not reported
2014	Agarwal et al. [53]	22/M	Cytologic examination	20 U/L	AML, M2, diagnosed 2 months after leukemic effusion	Induction araC + Daunorubicin, second induction with HAM, consolidation with HiDAC	Bone marrow remission on day 47 of HAM; died during the neutropenic phase
2015	Lokireddy et al. [54]	74/M	Cytologic examination, flow cytometry	Not reported	AML, newly diagnosed	Induction araC + Daunorubicin	Clearance of blast cells in pleural fluid
2015	Suharti et al. [55]	46/F	Cytologic examination	Not reported	AML, M0, newly diagnosed	Induction araC + Daunorubicin	Refused further chemotherapy and home hospice
**2020**	**Present study**	**55/M**	**Cytologic examination**	**42 U/L**	**AML, M4, newly diagnosed**	**3 + 7 induction with Idarubicin + araC**	**Effusion resolved without recurrence, patient died of septic shock about 4 months after diagnosis**

Abbreviations: BM = Bone marrow; COAP = Cyclophosphamide, Vincristine, Cytarabine, and Prednisone; araC = Cytarabine; CR = Complete remission; HSCT = Hematopoietic stem cell transplant; APL = Acute promyelocytic leukemia; ATRA = All *trans* retinoic acid; FLAG-IDA regimen = Fludarabine, Cytarabine, Idarubicin and G-CSF; MDS = Myelodysplastic syndrome; CMMoL = Chronic myelomonocytic leukemia; EMA protocol = Etoposide, Mitoxantrone, and Cytarabine; ICH = Intracerebral hemorrhage; PV = Polycythemia vera; BIDFA = twice-daily Fudarabine and Cytarabine; HAM = High-dose Cytarabine and Mitoxantrone.

a Only studies with full-text or abstract in English available from PubMed were included.

b Negative finding from cytologic examination and flow cytometry.

Predisposing risk factors associated with extramedullary involvement include monocytic or myelomonocytic differentiation (French-American-British [FAB] subtypes M4/M5), chromosomal abnormalities such as t(8:21) and inv(16), and expression of T cell markers including CD2, CD4, and CD7 [6,19,20]. Adhesion molecules, including CD15 and CD56, are believed to play a crucial role in the adhesion of leukemic cells to interstitial tissues [21]. In this case, high pleural ADA levels might be attributed to excessive extramedullary proliferation of monocytic leukemic cells.

The incidence of tuberculosis is twofold higher in patients with hematological malignancies when compared to that in the general population [22]. Both Gupta et al. [23] and Chen et al. [22] reported significantly higher incidences of tuberculosis disease among patients with AML than among those with other subtypes of hematological malignancies, at 6.3% (*n* = 95) and 2.87% (*n* = 1,011), respectively. The main risk factors associated with the development of tuberculosis included reduced immunity due to the primary hematological disease, age ≥50 years, and treatment with cytotoxic chemotherapy or steroids. Definitive diagnosis of *M. tuberculosis* infection is based on the clinical signs and symptoms as well as positive sputum and/or tissue culture(s); these methods can be very time consuming. As such, ADA in the pleural fluid has been used since 1983 [24] to facilitate early diagnosis of tuberculous pleural effusion; currently, this test is in wide use and has notably high sensitivity and specificity [25]. The ADA cut-off level typically considered in regions with high disease prevalence is 40 IU/L. Reportedly, high-ADA level in the pleural fluid is associated with a higher probability of TPE; furthermore, the diagnostic accuracy of ADA in TPE was influenced by age [26]. ADA level in the pleural fluid of 40 IU/L in a 55-year-old patient indicated the possibility of TPE with 60% certainty [26]. The ADA level in our 55-year-old patient was 42 IU/L. Therefore, it is possible that the evidence is not strong enough to consider TPE as a differential diagnosis in our patient. However, immunosuppressed patients may reportedly have significantly lower ADA activity [27]. Indeed, this case suggests that ADA levels detected in pleural effusions in patients with AML may introduce diagnostic complexities and lead to inappropriate therapy. Of note, this patient was unable to undergo more invasive procedures, including pleuroscopy or thoracoscopy with surgical biopsy, due to severe thrombocytopenia. Interferon-gamma release assays (IGRA) are in vitro blood tests to assess cell-mediated immune response by measuring the release of interferon-γ by T cells following the stimulation by antigens specific to the *M*. *tuberculosis* complex; these assays are now widely used to identify latent tuberculosis infections [28]. Because of the simplicity and noninvasive nature of IGRAs, these offer an attractive alternative to promptly diagnose TPE. However, two meta-analyses have suggested that commercial IGRAs that use either whole blood or pleural fluid have poor diagnostic accuracy in patients with suspected TPE [29,30].

Although the diagnosis of leukemic pleural effusion resulted from direct cytologic examination in our patient and in most of the previous case reports (Table 2), cytopathology typically plays a limited role in most cases of hematologic malignancy due to its low diagnostic potential, reported at 2.7% by Cakir et al. [31] and at 1.58% by Johnston [32]. To prevent misdiagnosis, clinicians need to be aware of this atypical and rare presentation of AML. To improve the diagnostic yield, cytogenetic studies might be considered as a routine component of the pleural fluid analysis in patients diagnosed with AML and presenting with a pleural effusion [33].

Reportedly, leukemic effusion typically resolved after chemotherapy (Table 2). A case report described a patient with newly diagnosed AML in whom complete remission was achieved with first induction chemotherapy; however, the effusions did not resolve. The effusions resolved after re-induction with high-dose Cytarabine [16]. In addition, Huang et al. reported a good relationship between peripheral blast counts and the magnitude of pleural effusion [18].

This is possibly the first case report of a patient diagnosed with AML with leukemic pleural effusion associated with a high pleural ADA level. Additional studies are needed to determine more precise relationships between AML-associated pleural effusions and pleural ADA levels.

## Appendix

**Table A1:** Vital signs before, during and after the induction chemotherapy

	Body temperature (°C)	Heart rate (per minute)	Respiratory rate (per minute)	Blood pressure (mm Hg)	SpO_2_ (%)	Oxygen use
One day before chemotherapy	36.9	110	18	95/68	97	Ambient air
Day 1 of chemotherapy	36.5	113	20	113/80	94	Ambient air
Day 2 of chemotherapy	35.9	108	20	120/80	96	Ambient air
Day 3 of chemotherapy	36.1	102	20	92/53	95	Ambient air
Day 4 of chemotherapy	36	88	18	92/61	97	Ambient air
Day 5 of chemotherapy	36.1	93	18	97/64	96	Ambient air
Day 6 of chemotherapy	35.8	98	18	93/58	96	Ambient air
Day 7 of chemotherapy	36	88	18	90/62	99	Ambient air
One Day after chemotherapy	36.2	97	18	93/56	98	Ambient air
